# Observer Perceptions of GenAI Use and Interpersonal Trust: The Roles of Warmth, Competence, and AI Literacy

**DOI:** 10.3390/bs16071221

**Published:** 2026-07-18

**Authors:** Zhen Zhang, Jiaying Geng, Chunhui Qi

**Affiliations:** 1 Faculty of Education, Henan Normal University, Xinxiang 453007, China; zhangzhen201912@htu.edu.cn (Z.Z.); 2410283164@stu.htu.edu.cn (J.G.); 2 Faculty of Education, Henan University, Kaifeng 475001, China

**Keywords:** generative AI use, interpersonal trust, perceived warmth, perceived competence, perceived AI literacy

## Abstract

Generative AI (GenAI) is widely used in learning and daily life, yet its associations with interpersonal trust remain unclear. Drawing on the Stereotype Content Model and social cognitive theory, this study (*N* = 406) examined whether perceived warmth and competence mediate the relationship between observers’ perceptions of a target individual’s GenAI use and interpersonal trust, and whether perceived AI literacy moderates this relationship. Results showed that GenAI use was negatively associated with interpersonal trust. Perceived warmth and competence statistically mediated this association: GenAI use was associated with lower perceived warmth and competence, which in turn were associated with lower interpersonal trust. Perceived AI literacy moderated the direct negative association. Specifically, this association was significant when perceived AI literacy was low but non-significant when high. However, perceived AI literacy did not moderate the indirect pathways. This study provides preliminary evidence for potential perceptual mechanisms and boundary conditions associated with GenAI use and interpersonal trust, offering insights for balancing efficiency and trust in human-AI collaboration.

## 1. Introduction

The widespread adoption of generative artificial intelligence (GenAI) is reshaping the psychological dynamics surrounding its use. A global survey of 23,218 university students by Ravšelj et al. (2025) found that 71% had used ChatGPT. Similarly, the 2026 Status of Higher Education Study conducted by Hrynowski and Marken (2026) reported that 57% of U.S. college students use AI tools at least weekly. In China, the vast majority of college students are using AI tools, primarily for research assistance and course learning (Li et al., 2024), a finding consistent with the international trend—artificial intelligence is increasingly becoming a core tool for enhancing efficiency in academic work, particularly in tasks such as content generation and text revision (Sebesta, 2025). The widespread use of artificial intelligence has attracted significant attention at the national level. For instance, China has issued the “AI + Education” Action Plan, which states the need to “guide students to scientifically understand and reasonably utilize intelligent technologies, enhance students’ AI literacy, stimulate their curiosity, cultivate innovative thinking, and improve their cognitive thinking and ability to solve complex problems” (Ministry of Education et al., 2026).

Public attitudes toward college students’ use of AI are not uniform, and some scholars have expressed potential concerns, including worries that users may become overly dependent on AI, leading to the degradation of independent thinking abilities (Q. Liu et al., 2024; Li et al., 2024), unclear academic ethics boundaries and risks of plagiarism (Gu, 2026; Li et al., 2024), as well as the unreliability of AI-generated information that may mislead learning (Li et al., 2024). These concerns may extend beyond abstract risk assessment to shape interpersonal perceptions of AI users, potentially because individuals tend to hold negative attitudes toward the algorithm itself or the various issues that may arise from its use. Research on algorithm aversion has revealed people’s tendency to reject algorithmic advice (Dietvorst et al., 2015), and such negative attitudes toward AI systems may spill over to AI users (Dang & Liu, 2024). For instance, individuals who use AI are perceived as less human (Dang & Liu, 2024), less moral (Zhou et al., 2025), and are viewed as having lower competence (Reif et al., 2025) and lower reputational standing (Benegal et al., 2026). However, existing evidence largely stems from medical, consumer behavior, or human–AI collaboration contexts, and tends to focus on the dichotomous variable of “use versus non-use”, with less attention paid to AI use as a continuous dimension in higher education peer evaluation contexts. Among Chinese university students, although they hold relatively positive attitudes toward AI (Yu et al., 2026), whether AI use triggers negative interpersonal evaluations from observers, and whether such evaluations operate through social cognitive processes to influence trust, remains to be further examined.

To further elucidate the link between GenAI use and interpersonal trust, the present study draws on the Stereotype Content Model (SCM; Fiske et al., 2002), which posits that social perception of others revolves around warmth and competence. Prior research has examined how people evaluate AI or its users along these dimensions in human–AI interaction contexts (Samson & Zaleskiewicz, 2026). However, impression formation in the human–AI–human triad—particularly how university students evaluate classmates’ AI use–remains largely unexamined. Accordingly, this study investigates how the extent of AI use affects interpersonal trust through observers’ perceptions of the user’s warmth and competence.

Nevertheless, whether perceived AI literacy, as viewed by observers, can buffer the negative social evaluations associated with AI use remains insufficiently tested. If such a buffering effect holds, it would suggest that enhancing one’s AI literacy is not only a matter of technical competence but may also shape one’s social image among peers. Thus, drawing on social cognitive perspectives, the present study seeks to examine how the extent of AI use affects interpersonal trust through observers’ perceptions of the user’s warmth and competence, and to test the moderating role of perceived AI literacy in this process.

### 1.1. GenAI Use and Interpersonal Trust

As artificial intelligence becomes increasingly embedded in higher education, both opportunities and concerns have emerged. On the one hand, extensive use of AI tools may raise questions about academic integrity (Abdallah et al., 2025; Crawford et al., 2023). On the other hand, AI has become an indispensable tool for university students, shown to reduce cognitive load and promote academic engagement (Dong, 2025; Abdallah et al., 2025). Given its varied roles and consequences, how AI use is conceptualized and measured matters for understanding its social implications. In this study, GenAI use was defined as the extent to which individuals employ generative AI tools to complete their academic work (adapted from Tang et al., 2022). AI use is not a static behavior but a dynamic continuum. Beyond its direct academic consequences, AI use also carries profound interpersonal implications that warrant empirical investigation.

Interpersonal trust is generally defined as the willingness to accept vulnerability and take risks based on positive expectations of another party’s actions, words, or decisions (McAllister, 1995; Mayer et al., 1995; Johnson-George & Swap, 1982). It encompasses both cross-situational, stable attitudinal components and context-dependent cognitive components, with situational factors exerting stronger explanatory power over trust levels (Scott, 1980). Accordingly, trust in AI users can be understood as a context-sensitive form of interpersonal trust, reflecting the situational nature of trust judgments (Johnson-George & Swap, 1982). Qi et al. (2024) further noted that trust is shifting from unidirectional human trust in AI toward mutual trust between humans and AI, extending from individual-AI dyads to multi-agent interactions involving multiple humans and AIs. Accumulating empirical evidence suggests that AI use can negatively affect interpersonal trust. Dang and Liu (2025) concluded that AI users tend to be perceived as less trustworthy by the public, reflecting widespread societal skepticism toward AI and those who adopt it.

Research across various domains has shown that AI use can undermine interpersonal trust in the user. In e-commerce, customers trust online retailers less when they learn that AI has been used to compose customer service emails (Hennighausen et al., 2025). In healthcare, patients perceive physicians who use AI for diagnosis or treatment as less competent and trustworthy (Reis et al., 2025). In education, students trust professors less when they believe AI has been used in grading or feedback (Schilke & Reimann, 2025). These findings collectively indicate that AI use can signal reduced trust in the user. However, most of these studies have focused on structured professional interactions. Less is known about how observers evaluate a target individual’s AI use and whether such evaluations shape interpersonal trust among university students in everyday academic contexts. Nevertheless, given that interpersonal trust judgments are inherently context-sensitive and that AI use signals reduced human agency across settings, a similar negative association is expected to emerge among university students.

Accordingly, we propose the following hypothesis:

**H1.**  *GenAI use is significantly negatively associated with interpersonal trust.*

### 1.2. The Mediating Role of Perceived Warmth and Competence

Given that human–computer trust operates through fundamental social cognitive mechanisms—specifically, perceptions of warmth and competence (Kulms & Kopp, 2018)—and that these two dimensions likewise serve as the universal foundation of interpersonal trust judgments (Cuddy et al., 2007), we therefore seek to explore whether social cognitive inferences about warmth and competence may mediate the relationship between observing others’ AI use and interpersonal trust toward those users.

Initial impressions of a person are primarily formed based on perceptions of warmth and competence. The Stereotype Content Model (SCM; Fiske et al., 2002) provides the theoretical framework for understanding how AI use influences interpersonal trust. SCM posits that individuals judge others along two fundamental dimensions of social perception: warmth (e.g., friendliness, sincerity, morality) and competence (e.g., intelligence, ambition, efficiency; Fiske et al., 2002; Zuo et al., 2020).

Empirical evidence suggests that AI is perceived as cold and mindless (Dang & Liu, 2024), and that using AI to communicate is perceived as less warm than human communication (Dang & Liu, 2025). When individuals choose to collaborate with AI rather than humans, observers tend to perceive the user as less warm and less competent. However, findings on whether AI use signals reduced competence remain mixed: Zhan et al. (2025) found that using intelligent machines reduces perceived warmth, whereas Reif et al. (2025) found that AI use lowers perceived competence. Most of this evidence stems from laboratory settings, with limited attention paid to peer evaluation contexts in higher education and to AI use as a continuous dimension. Thus, how AI use shapes perceptions of warmth and competence in peer evaluation contexts among university students remains unclear.

Warmth and competence perceptions are key predictors of trust formation (Wei et al., 2025). On this basis, when observers evaluate an AI user, they may first judge the user’s warmth and competence, and subsequently adjust their trust accordingly. Drawing on social cognitive perspectives and the Stereotype Content Model, the present study investigates how the extent of AI use influences interpersonal trust through observers’ perceptions of the user’s warmth and competence.

Based on the theoretical analysis above, this study proposes the following hypothesis:

**H2.**  *Perceived warmth and perceived competence mediate the relationship between GenAI use and interpersonal trust. Specifically, GenAI use is negatively associated with interpersonal trust through observers’ perceptions of the user’s warmth and competence.*

### 1.3. The Moderating Role of Perceived AI Literacy

Existing research indicates that AI use can enhance AI literacy levels (Wang et al., 2026); however, the relationship between the two is not simply one of positive determination. AI literacy is defined as an individual’s comprehensive ability to understand, use, and evaluate AI (Long & Magerko, 2020). In the present study, we focus on observers’ perceptions of a target individual’s AI literacy rather than the target’s self-reported literacy, consistent with our other-evaluation design. This provides the logical basis for treating perceived AI literacy as a moderator in this study.

Social Cognitive Theory (SCT) provides a useful framework for understanding how environmental and personal factors jointly shape behavior (Bandura, 1986). Extending this logic to the present study, observers’ perceptions of a target’s AI literacy may function as a contextual cue that moderates how AI use is interpreted and evaluated. Prior research on AI literacy has shown that higher AI literacy is associated with more effective and critical use of AI tools (Beckman et al., 2025; Chiu et al., 2024; Tully et al., 2024; Zheng et al., 2025). Drawing on this logic, we propose that when observers perceive a target as having higher AI literacy, they may interpret the target’s AI use as more appropriate and less problematic.

In summary, this study proposes Hypothesis 3:

**H3.**  *Perceived AI literacy significantly moderates the direct path from GenAI use to interpersonal trust. Specifically, the higher the level of Perceived AI literacy, the weaker the negative association between GenAI use and interpersonal trust.*

### 1.4. Overview of the Present Research

With the widespread application of generative AI in education and daily communication, its impact on interpersonal trust has gradually attracted attention. However, existing findings remain inconsistent: some studies have found that GenAI use enhances efficiency (Hümmer et al., 2025) and increases interpersonal trust (Purcell et al., 2025), whereas others have indicated that AI use may trigger negative evaluations (Reif et al., 2025).

**Figure 1:**
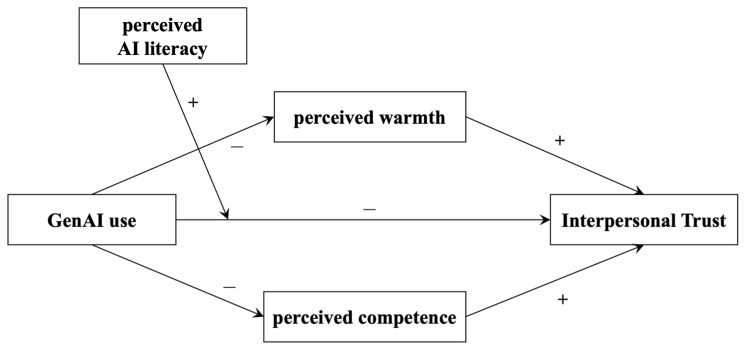
Conceptual model used in this research.

To explain this divergence, the present study draws on Stereotype Content Model (SCM; Fiske et al., 2002) to examine how GenAI use shapes observers’ perceptions of the user’s warmth and competence, which in turn influence interpersonal trust. At the same time, drawing on social cognitive theory, personal factors (e.g., perceived AI literacy) may moderate this process. Accordingly, this study proposes perceived AI literacy as a moderating variable affecting the aforementioned relationships (see Figure 1).

To systematically test the above hypotheses, the present study employed a recall method, asking participants to recall a specific classmate who had used AI and to focus on their specific characteristics, aiming to more comprehensively reveal the associations and boundary conditions through which GenAI use relates to interpersonal trust.

## 2. Materials and Methods

### 2.1. Participants and Procedure

During April 2026, a convenience sampling method was adopted to recruit participants. A total of 450 questionnaires were distributed via the Credamo platform to students from Henan Normal University and Henan Women’s Vocational College. After excluding 44 questionnaires that failed attention checks or exhibited straight-lining responses, 406 valid questionnaires were retained, yielding an effective response rate of 90.2%. Participants’ ages ranged from 17 to 23 years (M = 19.39, SD = 1.23). Among the participants, 88 were male (21.7%) and 318 were female (78.3%).

This study employed a questionnaire survey design. The independent variable was GenAI use, the mediating variables were perceived warmth and perceived competence, the moderating variable was perceived AI literacy, and the dependent variable was interpersonal trust. Questionnaires were distributed online via the Credamo platform to students from Henan Normal University and Henan Women’s Vocational College. The questionnaire consisted of three parts. The first part collected demographic information (gender, grade, major, and age). The second part included measures of GenAI use and perceived AI literacy. The third part comprised person perception scales, including measures of perceived warmth, perceived competence, and interpersonal trust. Participants completed the questionnaires sequentially in the order described.

Prior to completing the third part, participants read a set of instructions. This study adopted a recall method (Zhou et al., 2025), requiring participants to recall a specific classmate who had used AI and to write down the initials of that classmate’s name. This procedure was designed to focus participants’ attention on the specific characteristics of the target individual. Subsequently, participants completed the corresponding scales.

### 2.2. Methodology

All variables in this study were measured using established scales from international research. The original English scales were translated into Chinese and back-translated into English by two independent bilingual researchers following the standard forward-backward translation procedure, with discrepancies resolved through team discussion to ensure conceptual equivalence. According to Dawes (2008), 5-point and 7-point scales demonstrate high consistency in measurement invariance (Dawes, 2008). Therefore, all scales in this study were uniformly administered using a 5-point Likert scale (1 = strongly disagree, 5 = strongly agree). The scales used in this study are described below.

#### 2.2.1. AI Use Scale

The AI use scale was adapted from the scale developed by Tang et al. (2022), consisting of three items. Because the original scale was developed in a work context, we modified work-related terms (e.g., “position”, “job”) to “task completion” to reflect the change in context, and provided specific examples of generative AI. A representative item is: “This classmate uses generative AI (e.g., ChatGPT, Doubao, DeepSeek) to complete most of their academic tasks.” In this study, Cronbach’s α for this scale was 0.80.

#### 2.2.2. Perceived AI Literacy Scale

AI literacy was measured using the Perceived Artificial Intelligence Literacy Questionnaire (Grassini, 2024). The original scale contained six items (Appendix A). However, subsequent research indicated that item 2 (“I believe I can contribute to Generative AI projects”) showed inconsistency in its theoretical belonging and factor structure relative to the other items. Moreover, this item involves project development and deployment capabilities, which fall outside the scope of AI literacy (C. Liu et al., 2025). Following the approach of C. Liu et al. (2025), we deleted this item, resulting in a final 5-item version. Given that the present study adopted an other-evaluation (peer-rating) design, we converted the scale from a first-person to a third-person format. The scale was adapted using the same procedure as described above. Thus, this measure reflects perceived AI literacy of the target rather than the participant’s own AI literacy. A representative item is: “This classmate can understand the basic concepts of generative AI (e.g., ChatGPT, Doubao, DeepSeek).” In this study, Cronbach’s α for this scale was 0.80.

#### 2.2.3. Perceived Warmth Scale

Perceived warmth was measured using the warmth subscale developed by Fiske et al. (2002), consisting of six items. A representative item is: “I think this classmate is friendly.” In this study, Cronbach’s α for this scale was 0.92.

#### 2.2.4. Perceived Competence Scale

Perceived competence was measured using the competence subscale developed by Fiske et al. (2002), consisting of six items. The adaptation procedure was identical to that used for the warmth scale. A representative item is: “I think this classmate is competent.” In this study, Cronbach’s α for this scale was 0.91.

#### 2.2.5. Interpersonal Trust Scale

Interpersonal trust was measured using the trust scale developed by McAllister (1995), consisting of 11 items. A representative item is: “This classmate and I can freely share our personal ideas, feelings, and expectations.” The first five items measure affect-based trust (Cronbach’s α = 0.87), and the remaining six items measure cognition-based trust (Cronbach’s α = 0.80).

#### 2.2.6. Control Variables

Drawing on previous literature, we selected several control variables, including gender, grade, age, major, and relationship duration. Gender, grade, and age were included as standard demographic controls. Given that academic major may influence individuals’ AI use and perceived AI literacy, it was also controlled for. Furthermore, because this study employed a recall method requiring participants to evaluate a specific classmate, the duration of the relationship between the participant and the target individual was also included as a control variable.

### 2.3. Data Analysis

SPSS 27.0 and the PROCESS macro for SPSS were used to conduct common method bias testing, descriptive statistics, and correlation analysis. Mediation effects were tested using PROCESS Model 4, with 5000 bootstrap samples to estimate 95% confidence intervals. Moderated mediation effects were tested using PROCESS Model 8.

## 3. Results

### 3.1. Preliminary Analysis

Harman’s single-factor test was conducted to examine common method bias. The results showed that, without factor rotation, five factors with eigenvalues greater than 1 were extracted. The first factor accounted for 38.89% of the total variance, which is below the recommended threshold of 40%, indicating that common method bias was not a significant concern in this study.

### 3.2. Measurement Model

Given the inherent limitations of common method bias, particularly that Harman’s single-factor test serves only as a coarse diagnostic check and cannot statistically control for or partial out method effects, we adopted additional strategies to enhance confidence in our findings. First, we conducted a series of confirmatory factor analyses (CFA) to rigorously examine the discriminant validity of the five focal constructs: GenAI use, perceived AI literacy, perceived warmth, perceived competence, and interpersonal trust. As shown in Table 1, the hypothesized five-factor model demonstrated acceptable fit to the data: χ^2^/df = 2.84, CFI = 0.91, TLI = 0.90, RMSEA = 0.07, SRMR = 0.04. All factor loadings ranged from 0.62 to 0.87 (*ps* < 0.001), with composite reliability (CR) ranging from 0.80 to 0.92 and average variance extracted (AVE) ranging from 0.50 to 0.65, indicating adequate convergent validity. HTMT values for all construct pairs were below 0.85, supporting discriminant validity. Compared to alternative models, the five-factor model yielded the best fit (see Table 1) and was retained for subsequent analyses.

### 3.3. Descriptive Statistics and Correlation Analysis

Correlation analyses were conducted among the five variables: GenAI use, perceived AI literacy, perceived warmth, perceived competence, and interpersonal trust. The results are presented in Table 2. GenAI use was significantly positively correlated with perceived AI literacy, and significantly negatively correlated with perceived warmth, perceived competence, and interpersonal trust. Perceived AI literacy was significantly positively correlated with perceived competence and interpersonal trust, but was not significantly correlated with perceived warmth. Perceived warmth, perceived competence, and interpersonal trust were all significantly positively correlated with each other. Moreover, perceived warmth and perceived competence were also significantly positively correlated.

The means, standard deviations, and correlation coefficients for all variables are presented in Table 2. As shown in the correlation analysis, GenAI use was significantly negatively correlated with perceived warmth (*r* = −0.15, *p* < 0.01), perceived competence (*r* = −0.15, *p* < 0.01), and interpersonal trust (*r* = −0.19, *p* < 0.01). Perceived AI literacy was significantly positively correlated with GenAI use (*r* = 0.33, *p* < 0.01), perceived competence (*r* = 0.15, *p* < 0.01), and interpersonal trust (*r* = 0.15, *p* < 0.01). Perceived warmth was significantly positively correlated with perceived competence (*r* = 0.73, *p* < 0.01) and interpersonal trust (*r* = 0.71, *p* < 0.01). Perceived competence was also significantly positively correlated with interpersonal trust (*r* = 0.68, *p* < 0.01).

### 3.4. Testing the Mediating Effects of Perceived Warmth and Perceived Competence

**Figure 2:**
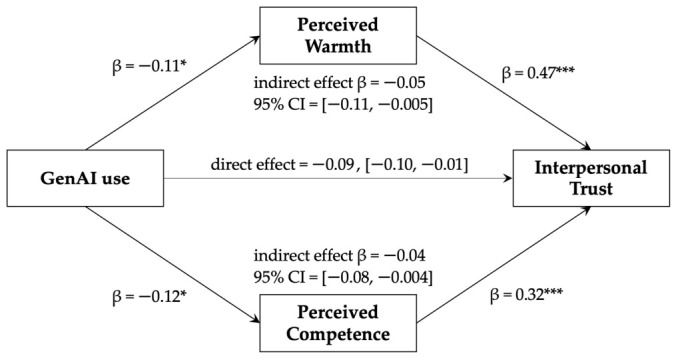
The parallel mediation model of perceived warmth and perceived competence. Note: All path coefficients are standardized; * *p* < 0.05, *** *p* < 0.001.

To test the mediating effects, we first conducted a linear regression analysis to examine the direct relationship between AI use and interpersonal trust. The results showed that AI use significantly and negatively predicted interpersonal trust (*β* = −0.19, *t* = −3.96, *p* < 0.001). We then conducted a mediation analysis using PROCESS Model 4 (Hayes, 2013), with gender, age, grade, major, and relationship duration included as control variables. The results are presented in Figure 2. After entering the mediating variables (perceived warmth and perceived competence), the negative predictive association of AI use on interpersonal trust remained significant (*β* = −0.09, *t* = −2.19, *p* < 0.05), but the effect size decreased substantially, indicating that the mediating variables explained most of the variance in the total association.

As shown in Figure 2, AI use significantly and negatively predicted perceived warmth (*β* = −0.11, *t* = −2.19, *p* < 0.05) and perceived competence (*β* = −0.12, *t* = −2.27, *p* < 0.05). Perceived warmth (*β* = 0.47, *t* = 9.71, *p* < 0.001) and perceived competence (*β* = 0.32, *t* = 6.68, *p* < 0.001) both significantly and positively predicted interpersonal trust. These results indicate that the mediating effects of perceived warmth and perceived competence are statistically significant, and that both play partial mediating roles in the relationship between AI use and interpersonal trust.

Bias-corrected percentile bootstrap method further showed that the mediating effect of perceived warmth was significant, with an indirect effect value of −0.05, 95% CI [−0.11, −0.01]. The mediating effect of perceived competence was also significant, with an indirect effect value of −0.04, 95% CI [−0.08, −0.004]. Neither confidence interval contained zero, confirming that both perceived warmth and perceived competence mediate the relationship between AI use and interpersonal trust. Thus, H1 and H2 were supported.

### 3.5. Testing Moderated Mediation Effects

Using PROCESS Model 8 (Hayes, 2013), we examined the mediating roles of perceived warmth and perceived competence in the relationship between AI use and interpersonal trust, as well as the moderating role of perceived AI literacy. With gender, grade, major, age, and relationship duration controlled for, bias-corrected percentile bootstrap method with 5000 resamples was used to estimate 95% confidence intervals.

The results are presented in Table 3. After simultaneously entering AI use, perceived warmth, and perceived competence into the regression equation, the direct association between GenAI use on interpersonal trust remained significant (*β* = −0.10, *t* = −2.98, *p* < 0.01). GenAI use significantly and negatively predicted perceived warmth (*β* = −0.15, *t* = −2.91, *p* < 0.01) and perceived competence (*β* = −0.18, *t* = −3.48, *p* < 0.001), whereas perceived warmth (*β* = 0.48, *t* = 10.20, *p* < 0.001) and perceived competence (*β* = 0.29, *t* = 6.06, *p* < 0.001) significantly and positively predicted interpersonal trust.

As for the moderating role of perceived AI literacy tested using PROCESS Model 8, the interaction term between GenAI use and perceived AI literacy did not significantly predict either perceived warmth or perceived competence. Specifically, the interaction effect on perceived warmth was (*β* = 0.04, *t* = 1.00, *p* = 0.32, 95% CI [−0.04, 0.11]), which contained zero. The interaction effect on perceived competence was (*β* = 0.05, *t* = 1.46, *p* = 0.15, 95% CI [−0.02, 0.12]), which also contained zero. These results indicate that the moderating effect of perceived AI literacy in the first stage of the mediation model was not supported.

However, the interaction term between GenAI use and perceived AI literacy significantly and positively predicted interpersonal trust (*β* = 0.13, *t* = 5.35, *p* < 0.001, 95% CI [0.08, 0.18]), with the confidence interval not containing zero. This finding suggests that perceived AI literacy moderates the direct path from GenAI use to interpersonal trust.

**Figure 3:**
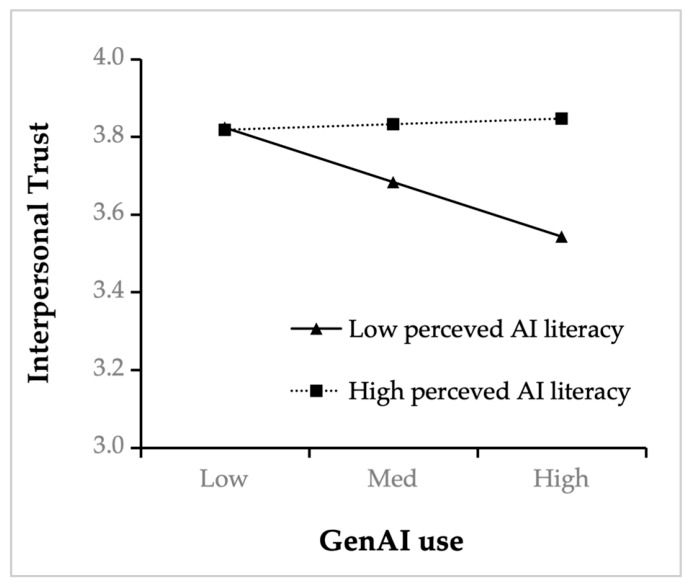
The moderating role of perceived AI literacy in the relationship between GenAI use and interpersonal trust (M ± 1 SD).

Simple slope analysis revealed that perceived AI literacy significantly moderated the relationship between GenAI use and interpersonal trust. Specifically, when perceived AI literacy was low, the direct effect of GenAI use on interpersonal trust was significantly negative (*β* = −0.23, *t* = −5.69, *p* < 0.001). When perceived AI literacy was at the average level, this effect remained significantly negative (*β* = −0.10, *t* = −3.00, *p* < 0.01). However, when perceived AI literacy was high, the effect was no longer significant (*β* = 0.03, *t* = 0.55, *p* = 0.57). Thus, H3 was supported. Moreover, the mediating effects of perceived warmth and competence remained significant in the moderated mediation model, further supporting H2. These findings indicate that perceived AI literacy was associated with a weaker negative association between GenAI use and interpersonal trust (see Figure 3).

In summary, as perceived AI literacy increased, the negative relationship gradually weakened and disappeared when perceived AI literacy was high.

Correspondingly, the bootstrap mediation analysis indicated that the index of moderated mediation was not significant. When perceived AI literacy was at the average level, the indirect effect of perceived warmth was not significant (indirect effect = −0.02, 95% CI [−0.02, 0.06]), whereas it was not significant at low or high levels of perceived AI literacy. For perceived competence, the mediating effect was significant at both low and high levels of perceived AI literacy. Specifically, at low perceived AI literacy, the indirect effect was −0.07, 95% CI [−0.11, −0.03]; at high perceived AI literacy, the indirect effect was −0.04, 95% CI [−0.07, −0.01]. Neither confidence interval contained zero. The indices of moderated mediation were not significant for either mediator perceived warmth (Index = 0.02, 95% CI [−0.02, 0.06]) or perceived competence (Index = 0.02, 95% CI [−0.01, 0.04]). These findings suggest that the role of perceived AI literacy primarily manifests in buffering the direct negative association between GenAI use and trust, rather than altering the mediating mechanisms of person perception.

## 4. Discussion

Drawing on a recall-based questionnaire survey (N = 406) and adopting an other-evaluation perspective, this study systematically investigated how GenAI use is associated with interpersonal trust, revealing the dual mediating roles of perceived warmth and perceived competence, and further examining the moderating role of perceived AI literacy. The results showed that GenAI use negatively associated with interpersonal trust, with perceived warmth and perceived competence playing partial mediating roles, and perceived AI literacy positively moderating the direct association between perceived AI use and interpersonal trust remained significant. In summary, GenAI use undermines interpersonal trust by reducing perceptions of warmth and competence, and this negative effect weakens as perceived AI literacy increases.

### 4.1. The Negative Association Between GenAI Use and Interpersonal Trust

Despite growing research on GenAI use, its association with interpersonal trust remains unclear. The present study addresses this gap by examining whether and how observers’ perceptions of others’ GenAI use relate to interpersonal trust.

The present study provides evidence for the key proposition that using AI is negatively associated with interpersonal trust. The results indicate that GenAI use significantly negatively associated with interpersonal trust; that is, observers tend to form negative impressions of others who use AI. This finding is consistent with prior research. For instance, Claessens et al. (2025) found that outsourcing tasks to AI reduces personality evaluations of the user due to perceived lack of effort, concern, and authenticity. Similarly, Spassova and Palmeira (2026) reported that using AI leads clients to be perceived as less competent and cold, thereby undermining their willingness to continue collaborating with a primary advisor.

Practically, this study offer implications for understanding the social consequences of GenAI use in educational contexts. First, high-level GenAI use may elicit negative interpersonal evaluations from observers, suggesting that users should be mindful of how their GenAI use might be perceived by peers. Second, the results highlight the importance of perceived AI literacy in buffering the negative association between GenAI use and interpersonal trust. When observers perceive that the user has a good understanding of AI’s strengths and limitations, they may view the user’s GenAI use as a reasonable strategy rather than a shortcut. These findings underscore the value of helping users articulate their GenAI use as an efficiency-enhancing practice, which may reduce the risk of being perceived as inauthentic or uncaring.

### 4.2. Perceived Warmth and Competence as Psychological Mechanisms Linking GenAI Use to Interpersonal Trust

This study examined the social cognitive mechanisms through which GenAI use associated with interpersonal trust. The moderated mediation results showed that GenAI use significantly and negatively predicted perceived warmth (*β* = −0.11, *p* < 0.05) and perceived competence (*β* = −0.12, *p* < 0.05), whereas perceived warmth (*β* = 0.47, *p* < 0.001) and perceived competence (*β* = 0.32, *p* < 0.001) significantly and positively predicted interpersonal trust, supporting partial mediation.

These findings are consistent with the Stereotype Content Model (Cuddy et al., 2007), supporting the applicability of the warmth-competence framework to understanding observers’ evaluations of AI users. Observers AI users as lower in both warmth and competence, and these perceptions in turn predicted reduced interpersonal trust. This pattern extends previous research on dehumanization (Dang & Liu, 2024) by showing that the negative perceptions associated with AI use may transfer to the user along both warmth and competence dimensions.

Notably, the effect sizes of perceived warmth and perceived competence showed comparable magnitudes, indicating that both warmth-related (e.g., sincerity) and competence-related (e.g., intelligence) traits are equally important in trust formation. This finding contributes to the broader literature by demonstrating that both dimensions shape interpersonal trust in educational peer evaluation contexts.

These findings also offer practical insights. When AI users demonstrate active and collaborative use, observers may form more favorable competence perceptions (Lee et al., 2026). This suggests that signaling appropriate use—rather than passive reliance—may help maintain positive perceptions of the user’s warmth and competence.

### 4.3. The Moderating Role of Perceived AI Literacy

How AI should be used and what competencies users need to master are central questions in current research. The present study found that perceived AI literacy significantly moderated the direct negative association between AI use and interpersonal trust (*β* = 0.13, *p* < 0.001). Simple slope analysis revealed that when perceived AI literacy was low, the negative association between AI use and interpersonal trust was significant (*β* = −0.23, *p* < 0.001); at average levels of perceived AI literacy, this association remained significant but weaker (*β* = −0.10, *p* < 0.01); when perceived AI literacy was high, the association was no longer significant (*β* = 0.03, *p* = 0.57). These findings suggest that perceived AI literacy is associated with a weaker negative association between AI use and trust: when observers perceive the user as having higher AI literacy, they may view the user’s AI use as more rational and less problematic. However, this null result should be interpreted with caution and requires replication in future research.

However, the moderated mediation analysis showed that perceived AI literacy did not significantly moderate the mediating pathways. Specifically, when perceived AI literacy was at the average level, the indirect effect of perceived warmth was significant but very small in magnitude, whereas it was not significant at low or high levels of perceived AI literacy. For perceived competence, the mediating effect was significant at both low and high levels of perceived AI literacy. Moreover, the indices of moderated mediation for both mediators were not significant. These findings suggest that the role of perceived AI literacy primarily manifests in moderating the direct negative association between AI use and trust, rather than altering the mediating pathways through perceived warmth and competence.

This finding carries important theoretical implications. When observers perceive the user as having higher perceived AI literacy, they may view the user as knowing how to use AI appropriately (Chiu et al., 2024); regardless of the extent of AI use, observers’ expectations of trust toward the user do not change significantly. In contrast, when perceived AI literacy is low, observers may rely more on surface behavioral cues—such as the mere fact of AI use—leading to stronger negative trust judgments. This study identifies perceived AI literacy as a boundary condition for the association between AI use and interpersonal trust, suggesting that perceived AI literacy may serve to reduce the negative social evaluations associated with AI use. These findings extend prior research on social evaluation of AI users by demonstrating that observers’ perceptions of the user’s AI competence can attenuate the negative association between GenAI use and interpersonal trust.

These findings also align with recent conceptualizations that distinguish between general AI literacy and critical thinking in AI use (Lau et al., 2026). Whereas AI literacy broadly refers to the ability to understand, use, and evaluate AI (Long & Magerko, 2020), critical thinking in AI use involves a more specific dispositional tendency to verify AI-generated information, understand how AI models work and where they fail, and reflect on the broader consequences of relying on AI (Lau et al., 2026). When observers perceive a target as high in AI literacy, they may infer that the target is likely to verify AI outputs, understand model limitations, and reflect on responsible use. These inferences may directly reduce trust concerns (i.e., “this person knows when to trust AI and when not to”), without necessarily altering perceptions of the target’s warmth or competence. This may help explain why perceived AI literacy buffered the direct negative association between AI use and trust but did not alter the mediating pathways through warmth and competence.

### 4.4. Limitations and Future Directions

First, regarding methodological limitations, this study employed a recall method in which participants were asked to recall a specific classmate who had used AI. This approach may introduce memory biases, as participants’ retrospective accounts may not fully reflect the target’s actual GenAI use. Additionally, participants may have selectively recalled classmates who were particularly salient—for example, those with whom they had prior positive or negative relationships—which could influence their evaluations of the target’s GenAI use. Furthermore, this study adopted a cross-sectional design, which precludes causal inferences among the variables. Future research could adopt experimental methods or field studies to validate the findings, and employ longitudinal designs to further examine the dynamic causal relationships and long-term mechanisms between GenAI use and interpersonal trust.

Second, regarding sample representativeness, the sample in this study was primarily recruited through an online platform, and participants were predominantly university students from two institutions in Henan Province, with females accounting for 78.3% of the sample. Thus, the sample representativeness is limited, and the generalizability of the findings requires further testing. Future research should extend to broader geographic regions, diverse institution types, various academic disciplines, and occupational populations to enhance the external validity of the conclusions.

Third, regarding theoretical mechanisms, this study, based on the Stereotype Content Model, identified the mediating roles of perceived warmth and perceived competence and examined the moderating effect of perceived AI literacy. The results showed that perceived AI literacy only attenuated the negative association between GenAI use and trust but did not significantly moderate the mediating pathways. Future research could introduce other mediating variables, such as perceived morality and perceived authenticity, which may serve as additional pathways through which GenAI use affects interpersonal trust. In addition, researchers could refine the multidimensional structure of perceived AI literacy to examine whether its different dimensions (e.g., technical understanding vs. ethical awareness) exert distinct moderating effects. The moderating roles of GenAI use styles (e.g., active collaboration vs. passive reliance) and task contexts also warrant further investigation.

Finally, with the rapid proliferation of generative AI, public attitudes toward GenAI use are undergoing dynamic changes. In contrast to the United States, where most students view using AI as a form of cheating (Golding et al., 2025), Chinese university students hold more positive and open attitudes toward generative AI use (Yu et al., 2026). While such cross-cultural differences are not the focus of the present study, they highlight the need for context-specific investigations. The cross-sectional design of this study failed to capture the long-term evolutionary trends of these attitudes. Future research could adopt cross-temporal comparisons or longitudinal designs to track the dynamic trajectories of Chinese university students’ AI use attitudes and trust judgments, and further explore how cultural factors shape the social evaluation mechanisms of GenAI use, thereby providing differentiated insights for AI governance across diverse cultural contexts.

## 5. Conclusions

The present study found that GenAI use is significantly negatively associated with interpersonal trust, with perceived warmth and perceived competence playing partial mediating roles. Further analysis indicated that this negative association is moderated by perceived AI literacy, but perceived AI literacy does not moderate the mediating pathways. Specifically, when perceived AI literacy was low, the negative association between GenAI use and interpersonal trust was significant; when perceived AI literacy was high, this negative association was no longer significant. Thus, higher perceived AI literacy is associated with a weaker negative association between AI use and trust. Moreover, perceived AI literacy moderated the direct path rather than the indirect pathways through perceived warmth and competence, suggesting that perceived AI literacy functions as a boundary condition for the interpersonal consequences of GenAI use rather than altering the social perception pathways. In summary, this study identifies the negative association between GenAI use and interpersonal trust and its mediating mechanisms, and clarifies the moderating role of perceived AI literacy, contributing to the understanding of how observers evaluate GenAI users and adjust their interpersonal trust in educational contexts.

## Figures and Tables

**Table 1:** Results of confirmatory factor analyses.

Model	χ^2^	df	χ^2^/df	CFI	TLI	RMSEA	SRMR
One-factor model (combining AU, AL, W, C, and T)	3119.52	434	7.19	0.67	0.64	0.12	0.10
Two-factor model (combining AU, AL; W, C, T)	2499.09	433	5.77	0.74	0.72	0.11	0.09
Three-factor model (combining AU, W, and C)	1967.84	431	4.57	0.81	0.79	0.09	0.07
Four-factor model (combining W and C)	1207.43	426	2.83	0.90	0.89	0.07	0.04
The hypothesized five-factor model	1201.97	424	2.82	0.91	0.90	0.07	0.04

Note: *N* = 406; CFI = comparative fit index; TLI = Tucker–Lewis index; RMSEA = root-mean-square error of approximation; SRMR = standardized root-mean-square residual; AU = GenAI use; AL = perceived AI literacy; W = perceived warmth; C = perceived competence; T = interpersonal trust.

**Table 2:** Descriptive statistics and correlations of variables (*N* = 406).

Variable	M	SD	1	2	3	4	5
1. AI use	3.34	0.90	—				
2. Perceived AI literacy	3.50	0.67	0.33 **	—			
3. Perceived Warmth	4.10	0.72	−0.15 **	0.08	—		
4. Perceived Competence	3.91	0.74	−0.15 **	0.15 **	0.73 **	—	
5. Interpersonal Trust	3.78	0.60	−0.19 **	0.15 **	0.71 **	0.68 **	—

Note: ** *p* < 0.01.

**Table 3:** The Moderated Mediating Effect of GenAI use on Interpersonal Trust.

Outcome Variable	Predictor Variable	*R* ^2^	*F*	*β*	*t*	95% CI
Perceived Warmth		0.08	4.21 ***			
	AI use			−0.15	−2.91 **	[−0.26, −0.05]
	Perceived AI literacy			0.16	3.18 **	[0.06, 0.27]
	AI use × Perceived AI literacy			0.04	1.00	[−0.04, 0.11]
Perceived Competence		0.10	5.54 ***			
	AI use			−0.18	−3.48 ***	[−0.28, −0.08]
	Perceived AI literacy			0.25	4.89 ***	[0.15, 0.35]
	AI use × Perceived AI literacy			0.05	1.46	[−0.02, 0.12]
Interpersonal Trust		0.51	46.19 ***			
	AI use			−0.10	−2.98 **	[−0.17, −0.04]
	Perceived Warmth			0.48	10.20 ***	[0.38, 0.57]
	Perceived Competence			0.29	6.06 ***	[0.19, 0.38]
	Perceived AI literacy			0.12	3.55 ***	[0.06, 0.19]
	AI use × Perceived AI literacy			0.13	5.35 ***	[0.08, 0.18]

Note: *N* = 406; All path coefficients are standardized; ** *p* < 0.01, *** *p* < 0.001.

## Data Availability

The data presented in this study are available on request from the corresponding author.

## Abbreviations

The following abbreviations are used in this manuscript:

AU	GenAI use
AL	perceived AI literacy
W	perceived warmth
C	perceived competence
T	interpersonal trust

## Appendix A

**Table A1:** Survey Instruments and Scale Items.

Construct	Questionnaire Item
GenAI Use	1. This student uses Generative AI (e.g., ChatGPT, Doubao, DeepSeek, etc.) to carry out most of their academic tasks. 2. This student spends most of their study time working with Generative AI (e.g., ChatGPT, Doubao, DeepSeek, etc.) to complete academic tasks. 3. This student works with Generative AI (e.g., ChatGPT, Doubao, DeepSeek, etc.) in making important academic decisions (e.g., method selection, content organization).
Perceived AI Literacy	4. This student clearly understands the basic principles of Generative AI (e.g., ChatGPT, Dou-bao, DeepSeek, etc.). 5. This student is able to evaluate the pros and cons of Generative AI (e.g., ChatGPT, Doubao, DeepSeek, etc.) in different scenarios. 6. This student has a good understanding of the latest development trends in the field of AI. 7. This student can discuss AI-related topics clearly and fluently with others. 8. This student can use Generative AI tools (e.g., ChatGPT, Doubao, DeepSeek, etc.) in innovative ways to solve problems.
Perceived Warmth	9. I think this student is friendly. 10. I think this student is warm and caring. 11. I think this student is warm. 12. I think this student is sincere. 13. I think this student is trustworthy. 14. I think this student is well-intentioned.
Perceived Competence	15. I think this student is capable for the task. 16. I think this student is prepared for this task. 17. I think this student is competent for this task. 18. I think this student will be efficient in completing the task. 19. I think this student is intelligent. 20. I think this student is skillful for this type of task.
Affect-based Trust	21. This student and I have a sharing relationship. We can both freely share our ideas, feelings, and hopes. 22. I can talk freely to this student about difficulties I am having in our collaboration and know that (s)he will want to listen and help. 23. I would feel a sense of loss if we terminated our collaboration after the task and could no longer work together. 24. If I shared my problems with this student, I know (s)he would respond constructively and caringly. 25. I would have to say that we have both made considerable emotional investments in this collaborative relationship.
Cognition-based Trust	26.I think this student approaches the task with professionalism and dedication. 27.Given this student’s past academic performance, I am confident (s)he has made adequate preparations for this task. 28.I can rely on this student to take the task seriously and not make my job more difficult by careless work. 29.Most people, even those who aren’t close friends of this student, would trust and respect him/her as a collaborator if they had the opportunity to work together. 30.Other study partners of mine who interact with this student consider him/her to be trustworthy. 31.If people knew this student better, they would be more concerned about and monitor his/her performance more closely. (Reverse-coded)
